# Permeability enhancement through hydraulic fracturing: laboratory measurements combining a 3D printed jacket and pore fluid over-pressure

**DOI:** 10.1038/s41598-019-49093-1

**Published:** 2019-08-29

**Authors:** Stephan Gehne, Philip M. Benson

**Affiliations:** Rock Mechanics Laboratory, School of Earth and Environmental sciences, University of Portsmouth, Portsmouth, PO1 3QL UK

**Keywords:** Natural hazards, Geophysics, Tectonics

## Abstract

The process of hydraulic fracture is well known in both natural (e.g. veining and mineralisation) and engineered environments (e.g. stimulating tight mudrocks and sandstones to boost their hydraulic properties). Here, we report a method and preliminary data that simulates both tensile fracture and fluid flow at elevated pressures. To achieve this we developed a sample assembly consisting of a cylindrical core drilled with an axial borehole encapsulated in a 3D printed jacket permitting fluid from the borehole to move through the freshly generated tensile fracture to a voluometer. The permeability of Nash Point Shale increases from a pre-fracture value of 10^−18^ to 10^−20^ m^2^ (1 microDarcy, μD to 0.01 μD) to 2 × 10^−15^ m^2^ (2 milliDarcy, mD) immediately after fracture (at 2.1 MPa confining pressure). Permeability is strongly dependent on confining pressure, decreasing to 0.25 × 10^−15^ m^2^ (0.25 mD) at 19 MPa confining pressure (approximately 800 m depth), and does not recover when confinement is removed. Using concomitant measurements of the radial strain as a proxy for fracture aperture, we conclude that the effective permeability is governed solely by the width of the developed cracks, revealed by post-test X-Ray Computed Tomography to be planar, extending radially from the central conduit.

## Introduction

Hydrofracturing is a common process in many areas of pure and applied geosciences, such as magma and dyke intrusions^1,2^, the development of mineral veins e.g.^3^ and the intentional hydraulic fracturing of impermeable rock formations in the hydrocarbon and geothermal energy industries e.g.^4–6^. In the exploitation of unconventional hydrocarbon resources, hydraulic fracturing has become a common – and controversial – technique for oil and gas reservoir stimulation to recover hydrocarbons from otherwise uneconomic formations of mudrock, which otherwise do not have sufficient permeability even though they contain trapped gas and oil within the trapped pockets of porosity^7–9^. Employing this technique has enabled one of the biggest and most influential developments in the energy sector in recent years; the exploitation of “unconventional reservoirs” loosely defined as sedimentary formations that cannot be produced by using a conventional well. By intentional stimulating new permeability, the extraction of hydrocarbons (both oil and gas) from unconventional reservoirs has transformed the US energy landscape resulting in the growth of total natural gas production^6^. Unlike conventional reservoirs (e.g. sandstone or carbonate formations) that are often buoyancy-driven deposits occurring as discrete accumulations in structural and/or stratigraphic traps^10^, unconventional reservoirs have a very low permeability (<20 × 10^−15^ m^2^; <20 mD, where 1 Darcy is commonly taken as approximately 1 × 10^−12^ m^2^) and are frequently composed of shale and occasionally, tight (cemented) sandstone and sometimes mixed with carbonate formations.

During hydraulic fracturing, a pore fluid is pumped into a wellbore at rates higher than the radial fluid flow into the surrounding rock, which is a function of the permeability of the rock mass. This leads to a pressure build up inside the borehole until the pressure is sufficient to induce new fractures at the borehole wall or to re-open and further propagate any pre-existing fractures or discontinuities. Fractures will extend until the rate of fluid loss into the formation exceeds the pumping rate^11^ and in doing so, create a high-conductivity pathway and a larger surface area in contact with the reservoir in order to extract trapped *in-situ* pore fluids. The first hydraulic fracturing treatment experiments for stimulation were performed in the Hugoton gas field in Kansas in 1947 by Stanolind Oil. In 1949, Halliburton Oil Well Cementing Company performed the first commercial fracturing treatment in Oklahoma and Texas: within the first year, some 330 wells were treated, increasing production by 75%. During the 1950’s, the application of hydraulic fracturing increased rapidly to up to 3,000 wells per month e.g.^12^.

The key aim of hydraulic fracturing is therefore to enhance extraction and flow rates through the generation of hydraulic fractures. Fracture conductivity, defined as the product of fracture permeability and fracture aperture, is a key indicator of this, used to evaluate the effectiveness of fracturing and to evaluate long term production^13^. However, due to the inherent inability to evaluate and ‘see’ the formation, obtaining geophysical data (e.g. rock strength, permeability, fracture toughness, anisotropy) and understanding its evolution with time is difficult, as the process necessarily requires a “trial and error” approach where by the enhanced permeability is only evident after the (irreversible) method has been carried out. Notwithstanding the use of various wellbore tools and core-plugs for spot measurement, various models have therefore been developed to understand the link between the fracture process and the generated permeability. Numerical models to describe the fluid flow through fractures perform well under certain conditions, however they cannot be applied universally without modification as the characteristics of the permeability evolution is modified under loading conditions specific to formation and rock type^14^. This challenge is particularly important for estimating permeability, and its variation with pressure and stress. To address this, laboratory measurements of fracture conductivity under controlled conditions in the laboratory have been reported, including the permeability of artificial fractures (sawn or split samples) and the effect of proppants^15–21^. To better mimic the typical borehole scenario, Bernier *et al*.^18^ conducted hydraulic fracture tests on hollow samples within a triaxial device. They observed 4–5 order of magnitude increases in permeability, rising to 10^−15^ m^2^ (1 mD) from initial values between 10^−19^ and 10^−22^ m^2^ (0.1 μD–0.1 nD). Similar work but also including Acoustic Emission (AE), the laboratory proxy to tectonic seismic activity, has further expanded on the links between new fracture development and the energy budget using mechanical borehole pressurisation via a rubber liner^5^ and via direct water injection^22^.

Although studies such as these have linked fracture energy to the stress and other conditions, measuring the permeability of a newly generated fracture in the same experiment (and therefore minimising sample preparation effects such as sawcuts/split samples) are not common. This is important information, as the permeability of an aligned fracture (without fracture offset) increases by approximately 3 orders of magnitude compared to the virgin shale (matrix) permeability^21^. The permeability increases further, by about 1–2 orders of magnitude, when fractures are displaced, and when some form of proppant (such as microbeads) is included, the permeability increases still further, by another 2–4 orders of magnitude. This large increase in permeability is supported by electrical conductivity data using samples of Barnett Shale. Here, unpropped, aligned fractures provided a conductive path some 3 orders of magnitude lower than fractures using a proppant^21^, which was additionally found to be dependent on proppant dimension and concentration. In addition to aperture, fracture permeability is known to be a function of rock compressibility and roughness, as well as the effective stress present in the rock mass^13,20^, adding to the complexity of the system. The impact of effective stress and roughness on fracture permeability suggests that jointed rock is more sensitive to pressure than the intact rock e.g.^15^. And, whilst the use of a proppant is common in the engineered environment, any measure that leads to an increase in fracture offset will enhance permeability^19^.

The estimation of the newly generated permeability, whether generated due to natural of an engineered process, poses a challenge. Although the permeability of rocks have been measured across a range of simulated depths in many studies an using many different methods e.g.^23–25^, including the use of samples with pre-exiting joints or fracture sets, linking these data to the large scale process remains challenging. Partly this is due to the different scale – a common challenge to all laboratory approaches – but also due to the difficulty in measuring the fluid permeability of a freshly generated fracture in tension due to the fluid overpressure, and without disturbing the crack (offset, aperture, etc.) in the process. In the field, the method is further complicated by the inability to positively link the newly generated permeability with burial pressure and with respect to the energy needed to generate and extend that fracture. These are important gaps in our knowledge, as better analysis of the data regularly collected *in-situ* (such as injection pressure and the local seismicity), would potentially allow a better spatial estimate of the network to be derived. And, although laboratory work has been performed to address some of these links, these studies often involve either artificial fractures created by cutting the sample, or additional handling of the sample which risks changing the properties of the fracture.

There is an extensive literature dealing with the permeability of crustal rocks, including reservoirs rocks e.g.^17,23–25^. However, the majority of studies deal with fluid flow though intact materials, rather than through both the country rock and a newly-induced fracture (such as a simulated hydrofracture). In this study, we report a method that addresses some of the laboratory pitfalls above by hydraulically fracturing a low permeability shale sample using direct fluid overpressure, and then measuring the fluid flow through the fracture by means of a 3-D printed liner to guide fluid to a voluometer to record flow regardless of the fracture orientation. This allows the setup to record both the fracture process and the resulting permeability in the same set-up, as a function of effective pressure. As such, this approach does not require additional handling of the sample and minimises the changes of the fracture properties. Finally, we link these data to mechanical and acoustic properties using an embedded array of Acoustic Emission sensors to record and link the concomitant microseismicity to the fracture and fluid flow through the damage zone.

## Methods

We use Nash Point Shale (NPS) which has a low porosity and low permeability, both of which are key characteristics of the rocks found in unconventional reservoirs. Nash Point Shale (NPS) is part of the Porthkerry Member (Blue Lias Formation) from the Early Jurassic (190–200 ma) and was taken from outcrop on the south coast of Wales (UK). It is highly anisotropic (V_p_-anisotropy of 56%), has a porosity of 6.3% ± 0.7% and has a very low permeability (Argon gas) in the range of 1–2 × 10^−21^ m^2^ (1–2 nD). The rock consists of carbonates (≈60%), clay (≈20%) and silicates (≈20%) with a fine grained matrix of calcite cement and lithic fragments, predominantly quartz and chlorite. The average tensile strength (using the indirect Brazilian disc method) is 4.7 MPa parallel to bedding, and 8.8 MPa normal to bedding giving a strength anisotropy (max-min/mean) of 60% at ambient conditions.

**Figure 1:**
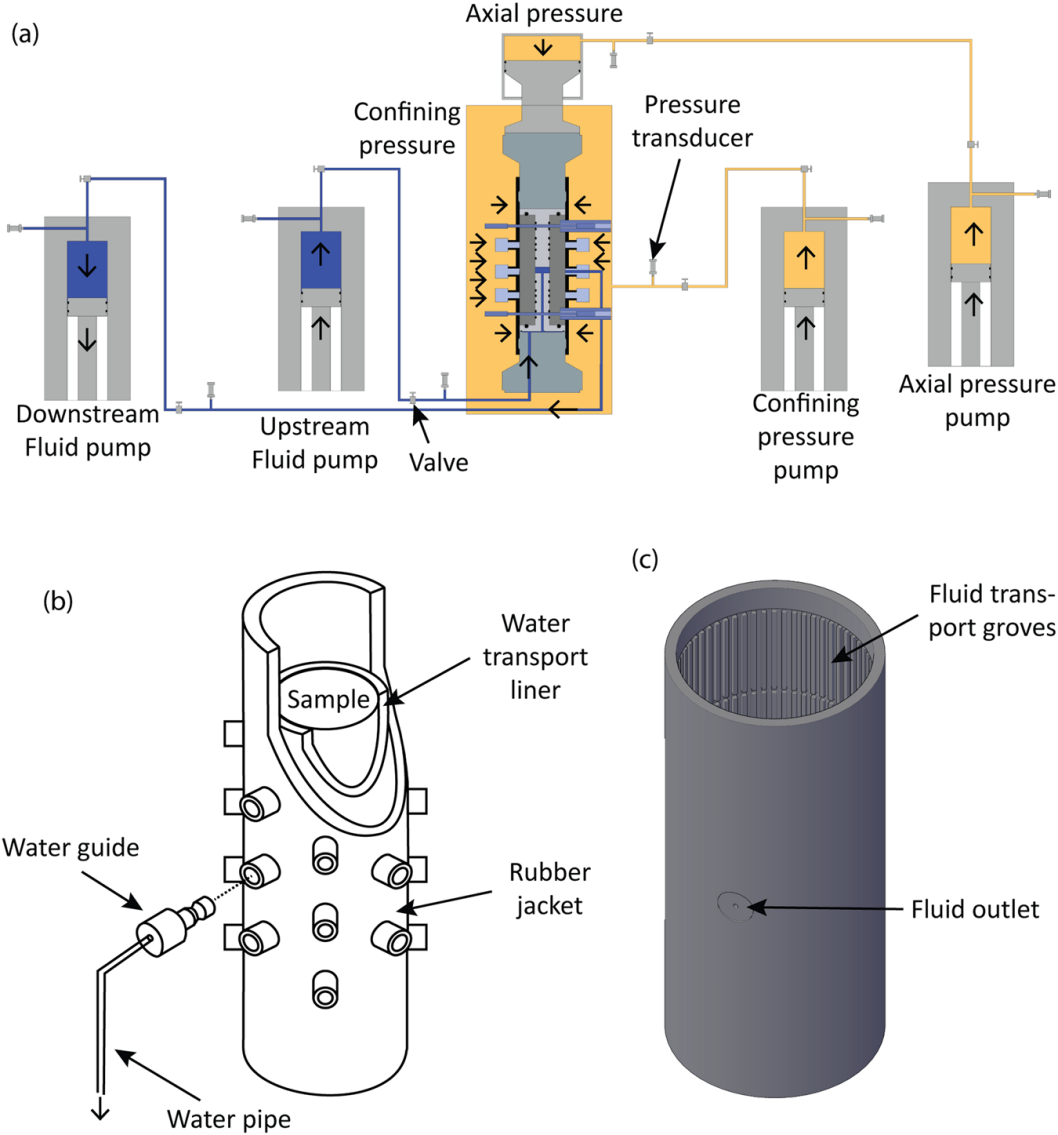
MHF permeability set up overview; (**a**) schematic of TRX set up for permeability measurements after hydraulic fracturing, (**b**) schematic of sample set up with water transport liner and rubber jacket and (**c**) 3D image of water transport liner.

**Figure 2:**
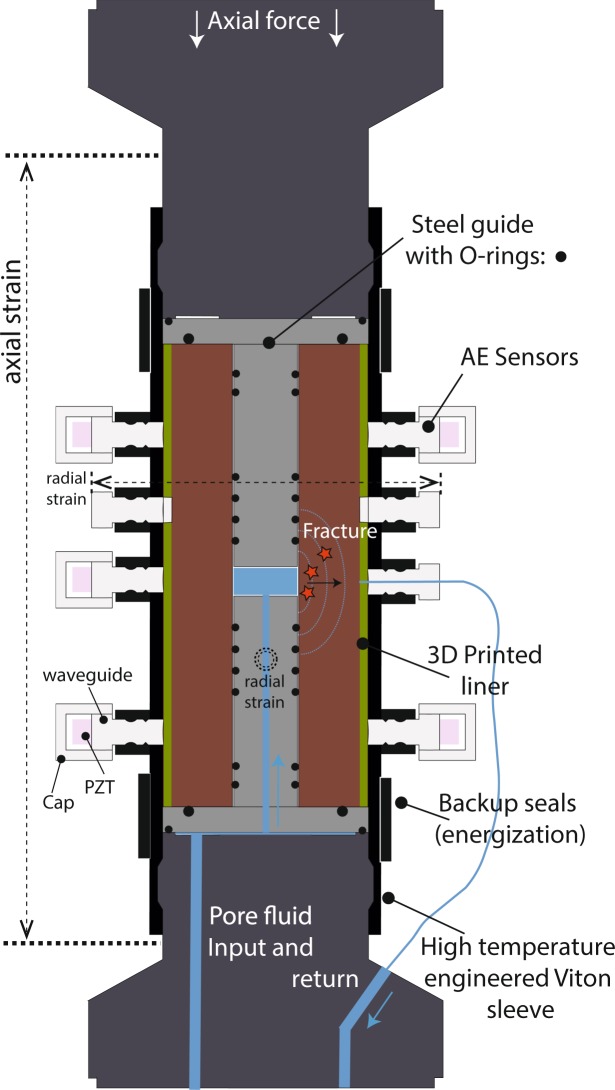
Detail of sample assembly in the triaxial cell. Radial strain is measured via internal LVDT cantilevers spaced at 90 degrees to each other (for clarity only 1 dimension is shown) and in direct contact with the sample though holes in the liner. Sealing is ensured via a multi-layered system of O-rings backed up by gaskets between the water guide and anvil using a small axial force. An external ‘energisation’ collar ensures initial sealing. A suite of AE sensors are embedded in the engineered nitrile jacket (FKM rubber) using a series of ports featuring integral O-ring and backup rings.

Experiments used an internally pressurised thick walled cylinder^26^ to both simulate the hydraulic fracturing architecture as close as possible, and also because this arrangement subjects the sample to a true tensile tangential stress near the conduit wall, causing the sample to fail under tension. Hydraulic fracturing was induced by pressurising the internal bore with high pressure syringe pumps, with the sample mounted in a conventional triaxial cell within a rubber jacket and 3D-printed water transport liner (Fig. 1). The apparatus incorporates a test chamber which can be pressurised with either dry nitrogen gas (for confining pressures to approximately 10 MPa), or heat transfer oil (Julabo Bath fluid Thermal HS) for confining pressures to 100 MPa; in this study heat transfer oil was used for pressurisation. Servo-controlled syringe pumps are used to apply axial stress via a piston-mounted pressure intensifier, which increases the 100 MPa pump pressure to a maximum 680 MPa axial stress across a nominal 36 mm diameter sample. A second syringe pump provides confining pressure, which is by-passed for low pressures. An additional servo-controlled syringe pump supplies high-pressure pore fluid (distilled water) to the bottom of the test sample, where the pore fluid circuit connects with a lower steel ‘packer’ inside the lower section of the borehole. This is a steel insert that directs pressurized fluid into a sealed section of the axially drilled conduit using a number of rubber O-rings (Fig. 2). The fluid then applies a uniform pressure over the sealed interval, initiating tensile fracture from a pre-defined zone within the sample bore. Using two packers thus allows a central sealed chamber to be defined in the sample, which can be lengthened or shortened by adding or removing O-rings. For the experiments reported here, a pressurised volume 10 mm in diameter and 19.2 mm in length was used. To ensure the drilling did not further induce fracture, the conduit was drilled first inside a large radial arm drilling machine, followed by the over-coring of the 36 mm (inside diameter) barrel to create the final specimen^26^.

Importantly, this arrangement does not require the use of a rubber lining inside the conduit so that the pressurized fluid is in direct contact with the rock, allowing the fluid to both pressurise the inner bore until failure, and then flow through the newly generated crack. The sample setup of rock specimen encased in a 2 mm thick liner (Fig. 1c) sits inside a 3 mm thick engineered rubber nitrile jacket^27^ fitted with up to 20 ports (Fig. 1b) to which AE sensors may be fixed to the sample whilst maintaining a seal. Fluid flow through the fracture, after hydraulic fracturing, was measured using the steady-state-flow technique. Pore fluid, on exiting the new fracture, is collected by the 3-D liner (Fig. 1c), which has an inner network of grooves that collect the fluid regardless of the orientation of the fracture, directing the fluid to a central port fitted with modified sensor waveguide (Figs 1b and 2; Supplementary Fig. S1). This steel plug includes a centre hole connected to a short length of flexible steel piping and then to a second pore water pump/voluometer (Fig. 1a), allowing water flow data to be directly collected from a new fracture without disturbing he sample, and thus simulating the larger scale *in-situ* scenario.

To measure radial strain, two pairs of modified steel inserts in contact with the sample through the liner are connected to a pair of linear variable displacement transducers to measure radial deformation across the sample diameter via a cantilever/calliper arrangement^26^. This is shown in Fig. 2, which also details the AE sensors and pore fluid circuit described earlier. In these experiments, the cantilevers were orientated at 90 degrees with respect to each other (shown in Fig. 2 as horizontal dotted lines for one pair, and a double dotted circle for the pair measuring the direction in the direction of the reader). This ensures that any fracture is never more than 45 degrees from the measurement direction. However, in these experiments, the natural bedding plane of the sample (vertical with respect to Fig. 2) was intentionally orientated normal to one set of cantilever; this plane of weakness meant that this cantilever recorded all of the resulting deformation whilst the set in-line recorded negligible deformation. Finally, a sealing system of O-rings and top/lower gaskets prevents pore fluid from shortcutting the system. To check, before the fluid-flow section of the experiment, pore pressures were monitored for ~1 hour to check for an increase that would suggest the higher pressure confining fluid leaking to the pore pressure circuit.

Experiments are performed by applying three consecutive loading phases to generate hydraulic fracturing^8,26^. Firstly an initial triaxial stress (σ_1_) and confining pressure (P_c_) is applied until the required conditions are established, controlled by setting a stress-rate increase on the pumps/volumometers. A ratio between axial stress (σ_1_) to confining pressure (P_c_) between 2:1 and 4:1 was used. This ensured that a positive triaxial condition is established where samples were firmly held, but without inducing any non-reversible strain due to axial stress, but with sufficient stress to prevent the subsequent injection fluid back-pressure from lifting the axial piston (the process of fluid-driven fracture necessarily requiring high fluid pressure injection that eventually exceeds the confining pressure, but not the axial stress, in order for the method to work properly). During the experiments, care was taken to ensure that the axial stress was kept to less than 30% of the peak compressional strength of the rock. Furthermore, AE activity was closely monitored during loading to check that this critical state is not approached. Secondly, to allow any seismic activity to decay to a background level, a minimum of 10 minutes was allowed before the pressurized fluid was injected. For this, distilled water was used to establish initial fluid pressure slightly lower than the confining pressure. Finally, in third stage, fluid pressure is increased by applying a constant flow rate until microscopic and macroscopic failure occurred. Here, a flow rate of 1 mL/min was used. All data in the first stage were recorded at high sampling rate (10 MHz for AE, 10 kHz for mechanical and pressure data) for later processing.

After fracture, a steady state flow is established by applying a differential fluid pressure between the sample conduit pressure, and the pressure in the printed liner. This is achieved using the two pore pumps independently, and then monitoring the pump volume change with time, so that they act as independent voluometers. An apparent permeability was calculated from this data as a function of effective pressure conditions up to 25 MPa via steady-state flow and the application of Darcy’s law. The average differential fluid pressure was kept constant at approximately 5 MPa, increasing the axial and confining stresses (set to hydrostat) in increments of 3–5 MPa, and returning to the initial value in larger steps of approximately 10 MPa. After each change in effective pressure, the pore-fluid system was allowed to equilibrate to the new pressure conditions and establish a new steady flow, which was assumed to be the case when volume in the upstream reservoir decreased at a similar flow rate as the downstream volume increased. This also confirmed that no leaks were present in the system. A minimum of 2 minutes was used to ensure stable flow rates and a steady-state flow through the sample before permeability measurements were taken over a duration of approximately 3 minutes. All mechanical/pressure data (axial stress, confining pressure, pore pressures and reservoir volumes) were recorded and logged continuously at 2 s intervals. Volume rate changes in the upstream (internal) and downstream (outside the conduit) reservoir were used to calculate the volume flow rate at each confining pressure stage. We estimate the fracture cross-sectional area, through with fluid exits the conduit, as the product of the length of the pressurised zone (19.2 mm: the vertical distance between the two O-rings at the centre of the setup in Fig. 2) and the crack opening displacement as recorded from changes in the radial dimension of the sample, multiplied by 2 as a pair of cracks are generated (Supplementary Fig. S2). Permeability is finally calculated from Darcy’s Law using the fluid volume flow rate Q, pressure gradient (P_u_-P_d_), and flow length L using the following expression^25,28^:

1 $${\kappa }=\frac{{Q}}{{A}}\frac{{\mu }\,\ast \,{L}}{({{P}}_{{u}}-{{P}}_{{d}})}$$

where: *κ* = permeability (m^2^),

*Q* = volume flow rate (flow velocity) (m^3^/s),

*A* = total fluid-flux area (m^2^); equal to crack opening displacement × the length of the pressurised zone (19.2 mm) × 2.

*P*_*u*_ = internal fluid pressure (Pa),

*P*_*d*_ = conduit external fluid pressure (Pa),

*μ* = fluid viscosity (8.9 E^−4^ Pa·s),

*L* = length of fracture (0.013 m) (inner radius – outer radius).

## Results

**Figure 3:**
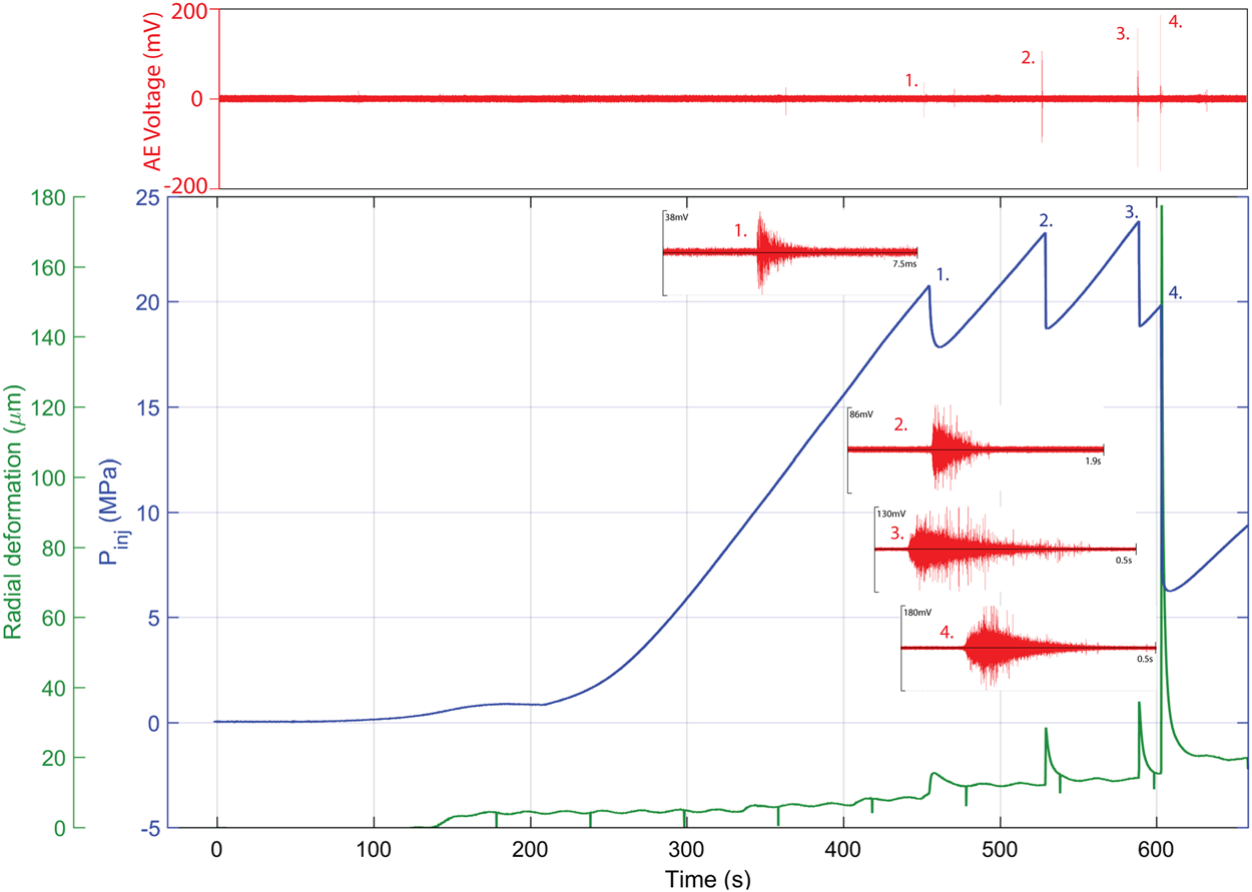
Experimental data record for the hydraulic fracturing (1^st^ experimental stage) of Nash Point Shale parallel to bedding at 8.4 MPa confining pressure. Pressure of the injecting fluid is plotted in blue, showing clearly the 4 discrete events constituting the hydrofracture. Waveforms 1 through 4 illustrate the changing character and amplitude of the fluid-mechanical response. In the initial stage 1, sharp onset and short, thought to larger amplitude in stages 2 and 3 with the latter of these having a longer wavetrain of more resonant character. Finally, the highest amplitude occurs during sample breakdown. Each stage I accompanied (green) by increasing radial strain jumps.

Typical results from the initial part of a standard hydraulic fracture experiment is presented in Fig. 3. Here, the initial fluid pressure setup is shown from 0 s to approximately 200 s (blue line) after which a constant fluid flow is set, pressurising the sample until failure. The failure is seen in this case as a sequence of four sharp fluid pressure decreases at approximately 450 s, 520 s, 590 s and 605 s. In each case, the pressure decrease is accompanies by a burst of AE (red), with each subsequent event of higher magnitude that the last, although the overall number and magnitude of recoded AE is low for these type of experiments. Early waveforms (Fig. 3-1) are low amplitude (38 mV), short, and with an impulsive onset, whereas by the end of the sequence, waveform are higher amplitude, up to 200 mV, and longer (500 ms) with an emergent onset often linked to fluid flow^29,30^ – which is a known mechanism in these experiments. Each pressure drop and AE swarm coincides with spikes in the radial deformation time-curve, which increase with every successive step up to a maximum of just under 180μm at the final pressure decrease. Subsequently, radial deformation decreases and stabilises at ≈20 μm ready for the fluid flow (permeability) measurement stage. Fractures tend to develop parallel to sub-parallel to the bedding planes^26^, and are planar and of uniform thickness between 60–120 μm.

**Figure 4:**
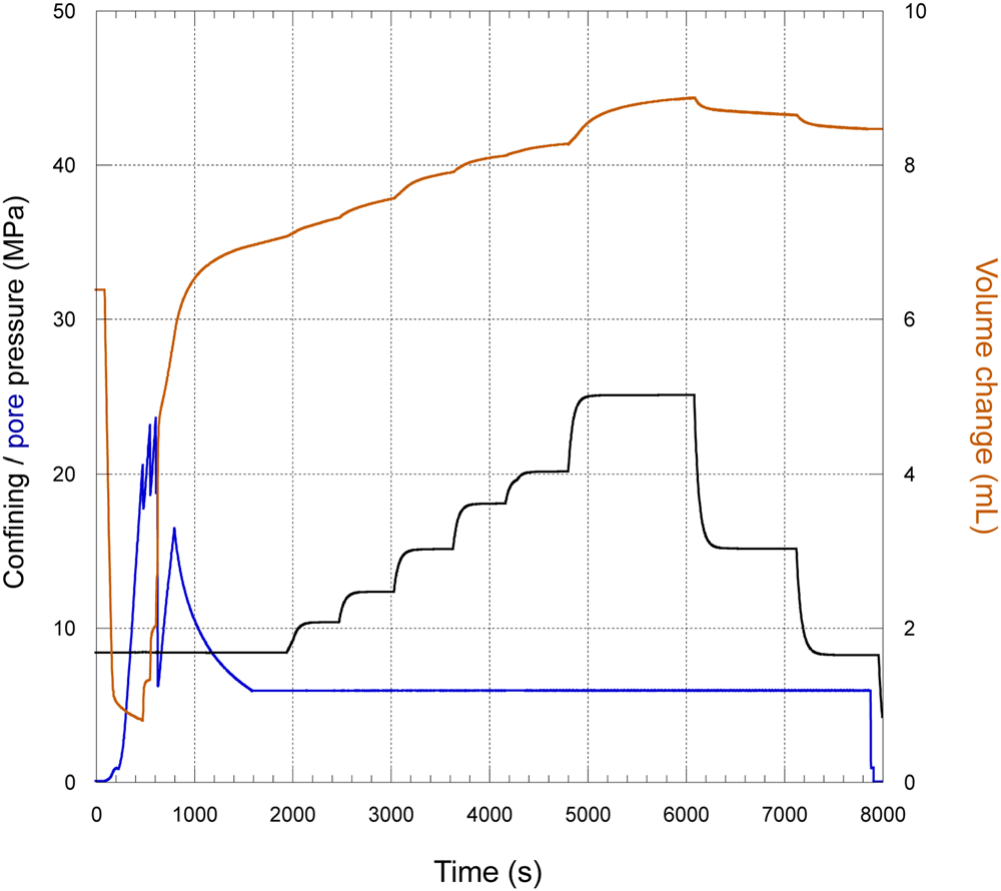
Laboratory data for the 2^nd^ (low speed) stage of the experiment (from 1600 s onwards) showing the pore voluometer change (orange) with time, superimposed with the step increase in confining pressure (black). During this stage, a constant pressure differential is maintained between borehole and outlet (liner) pressure to allow permeability to be calculated. Radial (aperture) data is taken manually during each phase as listed in Table 1.

**Figure 5:**
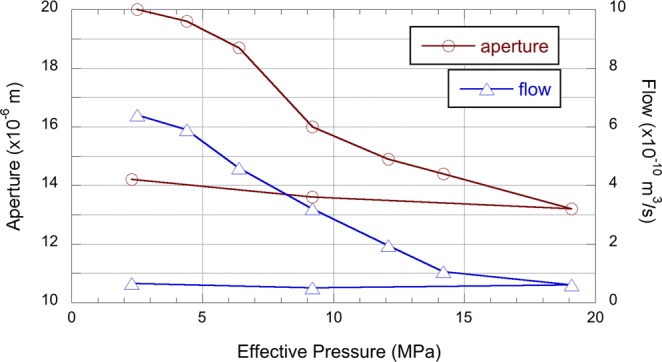
Aperture (dark red open circles; left hand axis) and flow rate (blue open triangles; right hand axis) data as a function of effective pressure. Both data show decreasing values that do not recover when the effective pressure is removed, with flow rate particular prone to this behaviour showing no recovery from 19 to 2.1 MPa.

With the initial part of the experiment complete, two freshly generated tensile fractures have been generated and monitored; this is verified post experiment by X-Ray Computed Tomography imaging (Supplementary Fig. S2). This particular shale often fractures with only a single tensile crack (Supplementary Fig. S3), thus benefitting from a post-test verification in cases where the fractures are not immediately obvious in hand specimen. The second part of the experiment, starting from approximately 1900 s (Fig. 3), measures changing fracture permeability for stepwise increases in effective pressures from 2.5 MPa to 25 MPa, followed by two recovery stages at 15 MPa and 8 MPa. After fracture, average volumometer change with time (Fig. 4, orange line) is used to calculate a flow rate (Fig. 5) during each step change in effective pressure (P_eff_) by increasing the confining pressure P_c_ (Fig. 4, black line) for constant pore pressure P_p_ (Fig. 4, blue line) via P_eff_ = P_c_ − P_p_. At each increase in Pc, an obvious jump in voluometer position is seen, followed by a period over which the fluid rate stabilises over several minutes until steady state is re-established. To calculate permeability, the measured volume flow rate is combined with radial deformation (Fig. 5), which approximates fracture aperture at each pressure stage. For the calculation of the total surface area of the fracture, a single rectangular fracture profile is assumed, defined by the crack opening as derived from radial deformation data, and the length of the pressurised zone (19.2 mm), summarised in Table 1.

**Table 1:** Results of permeability measurements after hydraulic fracturing of Nash Point Shale; P_c_ – confining pressure, P_eff_ – effective pressure.

Phase	Crack aperture (μm)	Cross section ‘A’ (m^2^)	P_eff_ (MPa)	P_c_ (MPa)	Flowrate (m^2^/s)	Permeability (m^2^)
1	20.0	7.68 E-07	2.5	8.4	6.40 E-10	1.93 E-15
2	19.6	7.53 E-07	4.4	10.3	5.90 E-10	1.83 E-15
3	18.7	7.18 E-07	6.4	12.3	4.45 E-10	1.43 E-15
4	16.0	6.14 E-07	9.2	15.1	3.20 E-10	1.21 E-15
5	14.9	5.72 E-07	12.1	18.0	1.88 E-10	7.60 E-16
6	14.4	5.53 E-07	14.2	20.1	1.03 E-10	4.31 E-16
7	13.2	5.07 E-07	19.1	25.0	2.50 E-11	2.51 E-16
8	13.6	5.22 E-07	9.2	15.1	2.00 E-11	2.13 E-16
9	14.2	5.45 E-07	2.3	8.2	2.40 E-11	2.42 E-16

As shown in Fig. 5, both the variation in crack aperture, and the flow rate, follow a very similar trend decreasing rapidly as pressure increased. The decrease in crack aperture is particularly pronounced, decreasing to approximately 15 μm with the application of an additional 10 MPa, after which the decrease is quasi linear to a minimum of approximately 13 μm at 19 MPa. The variation in flow rate is similar with a largely linear decrease with increasing effective pressure up until 15 MPa, then with less decrease in flow rate with the final step increase to approximately 0.6 × 10^−10^ m^3^/s at 19 MPa effective pressure. In both cases neither the aperture of the flow rate recovered with the removal of effective pressure, particularly flow rate.

**Figure 6:**
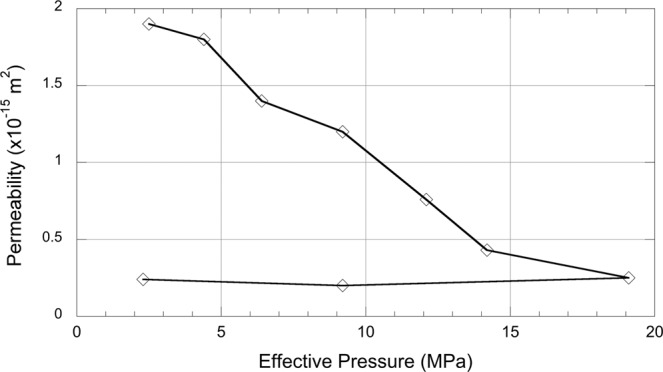
Crack permeability as a function of effective pressure for Nash Point Shale showing a steep, largely linear, decrease with increasing effective pressure to approximately 15 MPa, after which permeability does not decrease substantially. Permeability does not recover upon removal of the confining pressure.

Taking both aperture and fluid flow into account, and applying Eq. 1, allows an effective permeability of the crack to be calculated as a function of effective pressure (Fig. 6). An initial permeability of 1.9 × 10^−15^ m^2^ (1.9 mD) is calculated at an effective pressure of 2.1 MPa, which then decreases rapidly with increasing effective pressure in a quasi-linear fashion until an effective pressure of approximately 14 MPa, with the final measurement at 19 MPa decreasing by a smaller degree to the minimum permeability of 2.4 × 10^−16^ m^2^ (0.24 mD). As per the flow rate data, the permeability does not recover even after the effective pressure (confining pressure) is decreased to the starting value of 2.1 MPa.

## Discussion and Conclusions

In the engineered environment, a key aim of hydraulic fracturing is to enhance extraction and flow rates from otherwise unconventional reservoirs through the generation of hydraulic fractures. Related to this, a better understanding of the processes that develop tensile failure is of wider importance in natural phenomena, such as understanding fluid transport under active volcanoes and during the process of veining an mineralisation, both of which involve high pressure fluids. Fracture permeability depends on many factors such as fracture compressibility, fracture roughness and fracture surface offset as well as effective stress and rock strength^13,20^. Thus, it is important to understand the controls on fracture geometries; to this end, the ability to capture the evolution of hydraulic fracture conductivity under known laboratory conditions can provide key findings for reliable well performance analysis and optimizing fracturing design.

**Figure 7:**
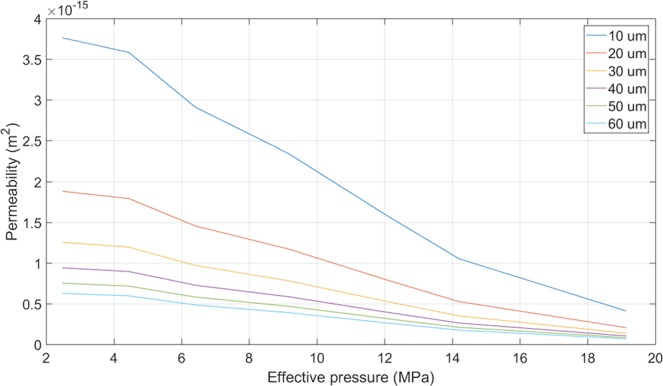
Calculated permeability of a slot of 19.3 mm height and 13 mm length (as per our setup), and with varying aperture between 10μm to 60μm as a function of effective pressure. The measured flow rate per pressure step, as measured in the reported experiment, was used to generate these data.

Here we have described a method to directly measure the fluid flow properties of a newly developed fracture allowing an *in-situ* flow rate as a function of confining pressure to be estimated without disturbing the sample via a saw-cut or similar setup. At an effective pressure of 2.5 MPa we measured a permeability of 1.9 × 10^−15^ m^2^ (2 mD) using an measured aperture of approximately 20μm. Although the permeability calculation uses a fixed crack length, defined here by the distance between the two inner O-rings (19.2 mm) and is therefore only an estimate of the fluid flow, it is unlikely fluid flow would not take the most energetically favourable route from the conduit to the sample edge. In addition, by adopting a holistic analysis considering the *change* in fluid flow, influences in external parameters may be related to changes in permeability. Compared to the initial permeability parallel to bedding for intact Nash Point Shale samples (10^−18^ m^2^ or ~1 μD), the bulk enhancement is approximately three orders of magnitude. This agrees well with permeability increases measured in other studies^18,20^. To further understand the variation of permeability with fracture aperture, Fig. 7 shows the effect of a range of initial crack apertures on permeability. As can be seen from (Eq. 1), an increase in the fracture area (fracture aperture), will lead to a decrease in overall permeability if other parameters are fixed. Likewise, even though a larger width will proportionally decrease the calculated permeability, the variation of decreasing permeability with increasing fracture area remains. For example, an approximate 20 μm crack leads to a permeability of just below 2 × 10^−15^ m^2^ (2 mD), and with increasing effective pressure over a range from 2 MPa to 19 MPa, decreases by one order of magnitude (Fig. 7). This shows a pressure dependency of fracture permeability, which ultimately controls the performance of the stimulation. This also suggests that the aperture is not the primary control on the apparent permeability of the induced fracture network, but the area of the fracture which would permit a larger fluid flow, as illustrated by our data whereby the permeability decreases with increasing pressure, despite a decreasing aperture.

**Figure 8:**
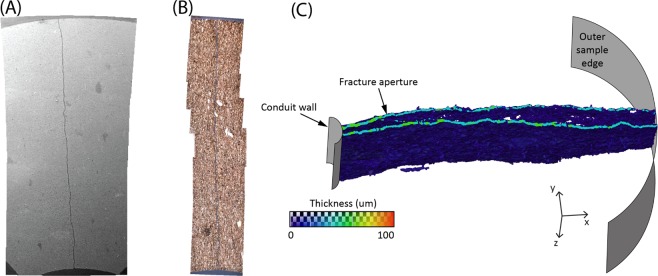
Photomicrographs of typical hydraulic fracture developed in (**A**) SEM and (**B**) microscope thin section. Thickness map of a hydraulic fracture in Nash Point Shale parallel to bedding (**C**) in a slice along the xy-plane through the fracture, further supports a fracture aperture in the 20–30 μm range.

It is likely that fracture permeability decreases under increasing compressive stress due to two factors: decreasing aperture (such as shown in Fig. 5) and an increasing resistance to flow through the fracture due to tortuosity effects^31^. Resistance to flow through the fracture may be caused by viscous drag of the fluid in the narrow openings between the two fracture surfaces, as well as the tortuosity of the flow path as fluid is diverted around asperities, which are in contact with the opposing fracture surface. Therefore, under increasing compression, aperture may decrease with a concomitant resistance to flow increasing due to smaller cross-sectional area, but also because the number of points and the area of contact between asperities of the opposing fracture surfaces increases, which leads to an increase in flow resistance due to the longer and more tortuous fluid path^15,25^. In many rocks, the closure of predominantly planar “crack” porosity is mostly complete by approximately 40 MPa e.g.^25^ however, in the experiments reported here, this appears to occur sooner, by approximately 14 MPa. We attribute this to the simpler and less tortuous pathways found in the fine grained mudrock used, as seen in post-test Scanning Election Microscopy, and X-Ray Computed Tomography (Fig. 8). The latter of these analysis provides direct data of fracture aperture – albeit after ample removal – also in the 20–30 μm range, and is therefore consistent with the aperture measured via the laboratory fluid flow dataset at elevated pressure. However, we also note that small scale tests of the sort presented in this study, and the small scale image analysis connected to them, ideally need to operate in tandem with larger scale experiments such as *in-situ* injection-producer methods common in the geothermal industry^32^ in order to better understand these complex, coupled, fluid-mechanics.

We conclude that, at low pressures, the contact area in freshly generated hydraulic fractures is relatively small, allowing for a relatively fast fracture closure. With increasing contact area, fracture closure is then inhibited by asperities, acting to decrease the closure rate and greatly reduce fluid flow ability of the fracture network. This is consistent with our data and also explains the observation by Kassis and Sondergeld^19^ that fracture offset is equally or even more effective in enhancing fracture permeability than proppants. Although the study reported here does not make use of proppants, we note that these type of fractures (mode-1) are particularly susceptible to crack closure, both by virtue of the fluid-driven mechanism that generates them, and by the later changing effective pressure conditions. This is further supported by the observation that the overall permeability is influenced by the tortuosity and fluid flow thought the fracture network, rather than the aperture. At the release of confining pressure, fracture permeability did not recover, leaving a permanent reduction of permeability. Such permeability hysteresis is a well reported phenomenon, and likely enhanced due to the high clay content of mudrocks^15,24^.

Finally, we conclude that fracture permeability is significantly influenced by not only the pressure used to drive the initial fracture, but by the subsequent pressure changes. From our experiment, we conclude that a key pressure level related to conditions found at approximately 1 km depth (around 25 MPa confining pressure and 5 MPa pore pressure) below which the pressures needed to keep fracture open increases significantly. Combined with the hysteresis effects, economic extraction of resources will need some form of proppant or fracture offset mechanism to ensure fracture permeability is maintained and permit fluid flow (extraction) over a protracted timescale.

## Supplementary information

Supplementary information

## References

[1] Rubin AM (1993). Tensile fracture of rock at high confining pressure: implications for dike propagation. Journal of Geophysical Research.

[2] Tuffen H, Dingwell D (2005). Fault textures in volcanic conduits: evidence for seismic trigger mechanisms during silicic eruptions. Bulletin of Volcanology.

[3] Gudmundsson A, Brenner SL (2001). How hydrofractures become arrested. Terra Nova.

[4] Bennion, D., Thomas, F. & Bietz, R. Low permeability gas reservoirs: problems, opportunities and solutions for drilling, completion, stimulation and production. *SPE Gas Technology Symposium*, 35577, 10.2118/35577-MS (1996).

[5] Vinciguerra S, Meredith PG, Hazzard J (2004). Experimental and modeling study of fluid pressure-driven fractures in Darley Dale sandstone. Geophysical Research Letters.

[6] Wang Q (2014). Natural gas from shale formation – The evolution, evidences and challenges of shale gas revolution in United States. Renewable and Sustainable Energy Reviews.

[7] Zoback M (1977). Laboratory hydraulic fracturing experiments in intact and pre-fractured rock. International Journal of Rock Mechanics and Mining Sciences & Geomechanics Abstracts.

[8] Stanchits S (2011). Fracturing of porous rock induced by fluid injection. Tectonophysics.

[9] Gandossi, L. An overview of hydraulic fracturing and other formation stimulation technologies for shale gas production. *European Commission Joint Research Centre Institute for energy and transport technical reports*, 26347, 10.2790/99937 (2013).

[10] Law B, Curtis J (2002). Introduction to unconventional petroleum systems. American Association of Petroleum Geologists.

[11] Reinicke A (2010). Hydraulic fracturing stimulation techniques and formation damage mechanisms—Implications from laboratory testing of tight sandstone-proppant systems. Chemie der Erde-Geochemistry.

[12] Montgomery CT, Smith MB (2010). Hydraulic fracturing: History of an enduring technology. Journal of Petroleum Technology.

[13] Tan Y (2018). Laboratory study of proppant on shale fracture permeability and compressibility. Fuel.

[14] Ma L (2016). Novel 3D centimetre-to nano-scale quantification of an organic-rich mudstone: The Carboniferous Bowland Shale, Northern England. Marine and Petroleum Geology.

[15] Kranz R (1979). The permeability of whole and jointed Barre granite. International Journal of Rock Mechanics and Mining Sciences & Geomechanics Abstracts.

[16] Nygård R (2006). Brittle-ductile transition, shear failure and leakage in shales and mudrocks. Marine and Petroleum Geology.

[17] Davy CA (2007). Permeability of macro-cracked argillite under confinement: gas and water testing. Physics and Chemistry of the Earth.

[18] Bernier, F. *et al*. Fractures and self-healing within the excavation disturbed zone in clays (SELFRAC). Final report to European Commission (Project FIKW-CT2001-00182) Preprint at, www.euridice.be/sites/default/files/scientific/SELFRAC%20final%20report.pdf (2007).

[19] Kassis, S. & Sondergeld, C. H. Fracture permeability of gas shale: Effect of roughness, fracture offset, proppant, and effective stress. *SPE International oil and gas conference and exhibition in China*, 131376, 10.2523/131376-MS (2010).

[20] Guo T (2013). Experimental study of fracture permeability for stimulated reservoir volume (SRV) in shale formation. Transport in porous media.

[21] Zhang, J. *et al*. Laboratory measurement of hydraulic fracture conductivities in the Barnett shale. *International Petroleum Technology Conference*, 16444, 10.2523/IPTC-16444-MS (2013).

[22] Goodfellow SD (2015). Hydraulic Fracture Energy Budget: Insights From the Laboratory. Geophysical Research Letters.

[23] David CJ (2018). KG²B, a collaborative benchmarking exercise for estimating the permeability of the Grimsel granodiorite – Part 1: measurements, pressure dependence and pore-fluid effects. Geophysical Journal International.

[24] Gehne S, Benson PM (2017). Permeability and permeability anisotropy in Crab Orchard sandstone: Experimental insights into spatio-temporal effects. Tectonophysics.

[25] Benson, P. M. Experimental study of void space, permeability and elastic anisotropy in crustal rocks under ambient and hydrostatic pressure. PhD Thesis, University College London. Preprint at, http://discovery.ucl.ac.uk/1446540 (2004).

[26] Gehne, S. A laboratory study of fluid-driven tensile fracturing in anisotropic rocks. PhD Thesis, University of Portsmouth. Preprint at, https://ethos.bl.uk/OrderDetails.do?uin=uk.bl.ethos.765705 (2018).

[27] Sammonds P (1999). Understanding the fundamental physics governing the evolution and dynamics of the Earth’s crust and ice sheets. Philosophical Transactions of the Royal Society of London A: Mathematical, Physical and Engineering Sciences.

[28] Jones, C., Meredith, P. & Ayling, M. R. An Experimental Study of Elastic Wave Propagation Anisotropy and Permeability Anisotropy in an Illitic Shale. *SPE/ISRM Rock Mechanics in Petroleum Engineering*, 47369, 10.2118/47369-MS (1998).

[29] Benson PM, Vinciguerra S, Meredith PG, Young RP (2010). Spatio-temporal evolution of coupled hydro-mechanical seismicity: A laboratory study. Earth and Planet Science Letters.

[30] Fazio M, Benson PM, Vinciguerra SV (2017). On the generation mechanisms of fluid-driven seismic signals related to volcano-tectonics. Geophysical Research Letters.

[31] Walsh J (1981). Effect of pore pressure and confining pressure on fracture permeability. International Journal of Rock Mechanics and Mining Sciences & Geomechanics Abstracts.

[32] Kneafsey, T. J. *et al*. An overview of the EGS Collab project: Field validation of coupled process modeling of fracturing and fluid Flow at the Sanford Underground Research Facility. 43rd Workshop on Geothermal Reservoir Engineering, Stanford University, paper SGP-TR-213. Preprint at, https://pangea.stanford.edu/ERE/pdf/IGAstandard/SGW/2018/Kneafsey.pdf (2018).

