# Supplementary material for: Permeability enhancement through hydraulic fracturing: laboratory measurements combining a 3D printed jacket and pore fluid over-pressure
Source: Sci Rep. 2019 Aug 29;9:12573. doi: 10.1038/s41598-019-49093-1 (PMC6715672; doi:10.1038/s41598-019-49093-1)
Supplement: Supplementary file 1 — Supplementary information [file 41598_2019_49093_MOESM1_ESM.docx]

**Supporting materials for:**

**Permeability enhancement through hydraulic fracturing: laboratory measurements combining a 3D printed jacket and pore fluid over-pressure**

Stephan Gehne and *Philip M Benson

Rock Mechanics Laboratory, School of Earth and Environmental sciences, University of Portsmouth, PO1 3QL, U.K.

Corresponding authors: Stephan Gehne ([stephan.gehne@port.ac.uk](mailto:stephan.gehne@port.ac.uk)) and Philip Benson ([philip.benson@port.ac.uk](mailto:philip.benson@port.ac.uk))

This supporting information contains three additional figures to support the above manuscript, consisting of:

**Supplemental Figure 1:** Collection of 4 plates illustrating (A) the concertina of sample, 3D printed sleeve, outer FKM-B rubber jacket isolating the confining medium. The waterguide is here place partially seated for illustrative purposes. Panel (B) shows the rubber jacket with a single sensor (as an example, for clarity) and the centre fluid-feed port; the 3D printed sleeve is shown to the right with water-guides installed. Panel (C) shows detail of the fit of the sleeve inside the FKM-B rubber jacket. Finally panel (D) is a top view of the assembly inside the rubber jacket, showing the backup sealing ‘gasket’ and internal port to feed water to the centrally-located injector to the pressurized zone of the sample (refer to Figure 2 of main text for schematic).

**Supplemental Figure 2:** Micro X-ray Computed Tomography image of sample 132 (from which the flow data was measured) indicating the primary twin fracture network.

**Supplemental Figure 3:** Micro X-ray Computed Tomography images indicating the fracture network (highlighted in red) developed in Nash Point Shale with the core axis drilled parallel to bedding, black lines indicate the background bedding orientation (after Gehne, 2018). Panels (a) to (f) indicate fracture generated at different confining pressures (Pc), as indicated.


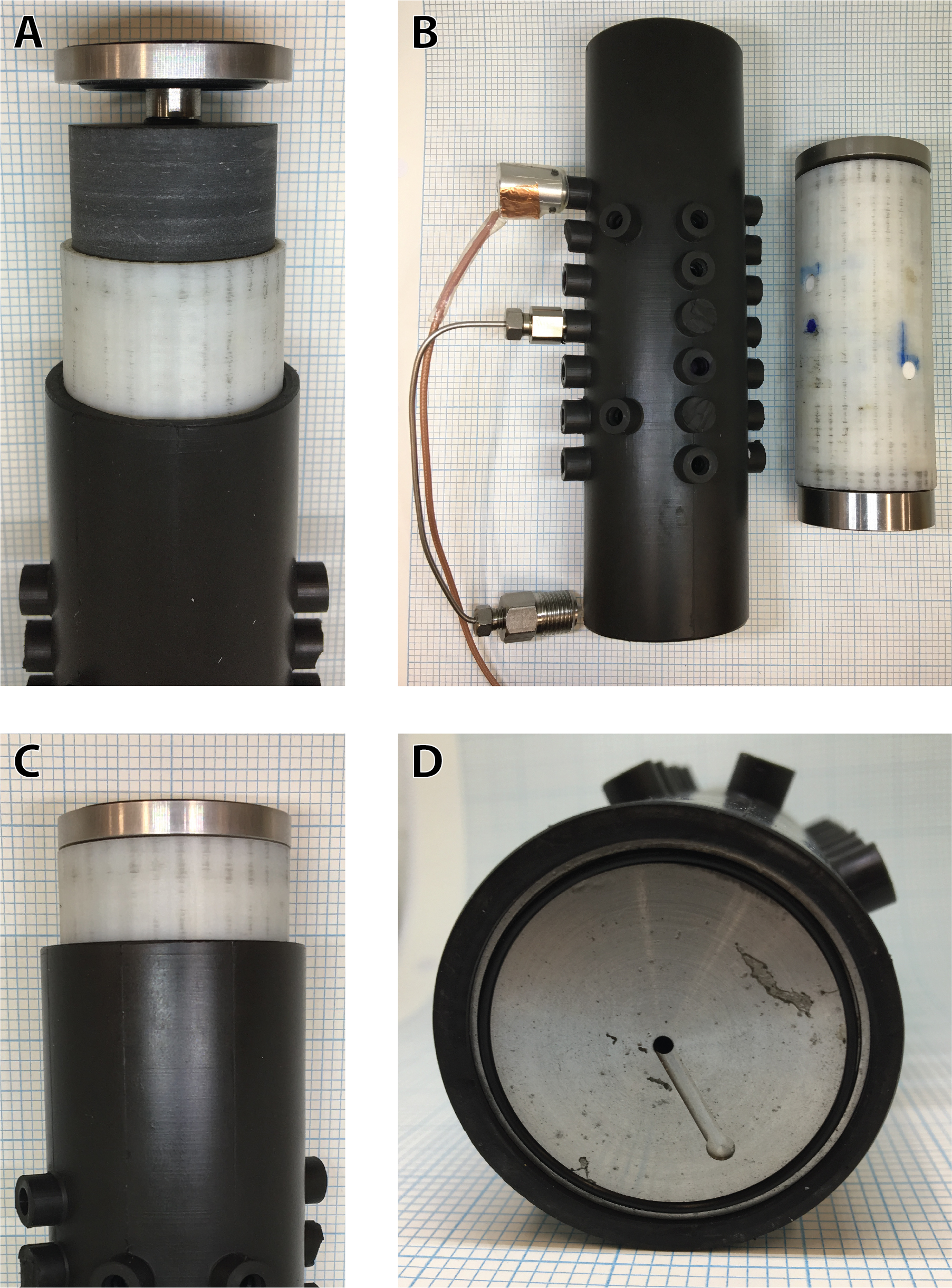


**Supplemental Figure 1:** Collection of 4 plates illustrating (A) the concertina of sample, 3D printed sleeve, outer FKM-B rubber jacket isolating the confining medium. The waterguide is here place partially seated for illustrative purposes. Panel (B) shows the rubber jacket with a single sensor (as an example, for clarity) and the centre fluid-feed port; the 3D printed sleeve is shown to the right with water-guides installed. Panel (C) shows detail of the fit of the sleeve inside the FKM-B rubber jacket. Finally panel (D) is a top view of the assembly inside the rubber jacket, showing the backup sealing ‘gasket’ and internal port to feed water to the centrally-located injector to the pressurized zone of the sample (refer to Figure 2 of main text for schematic).


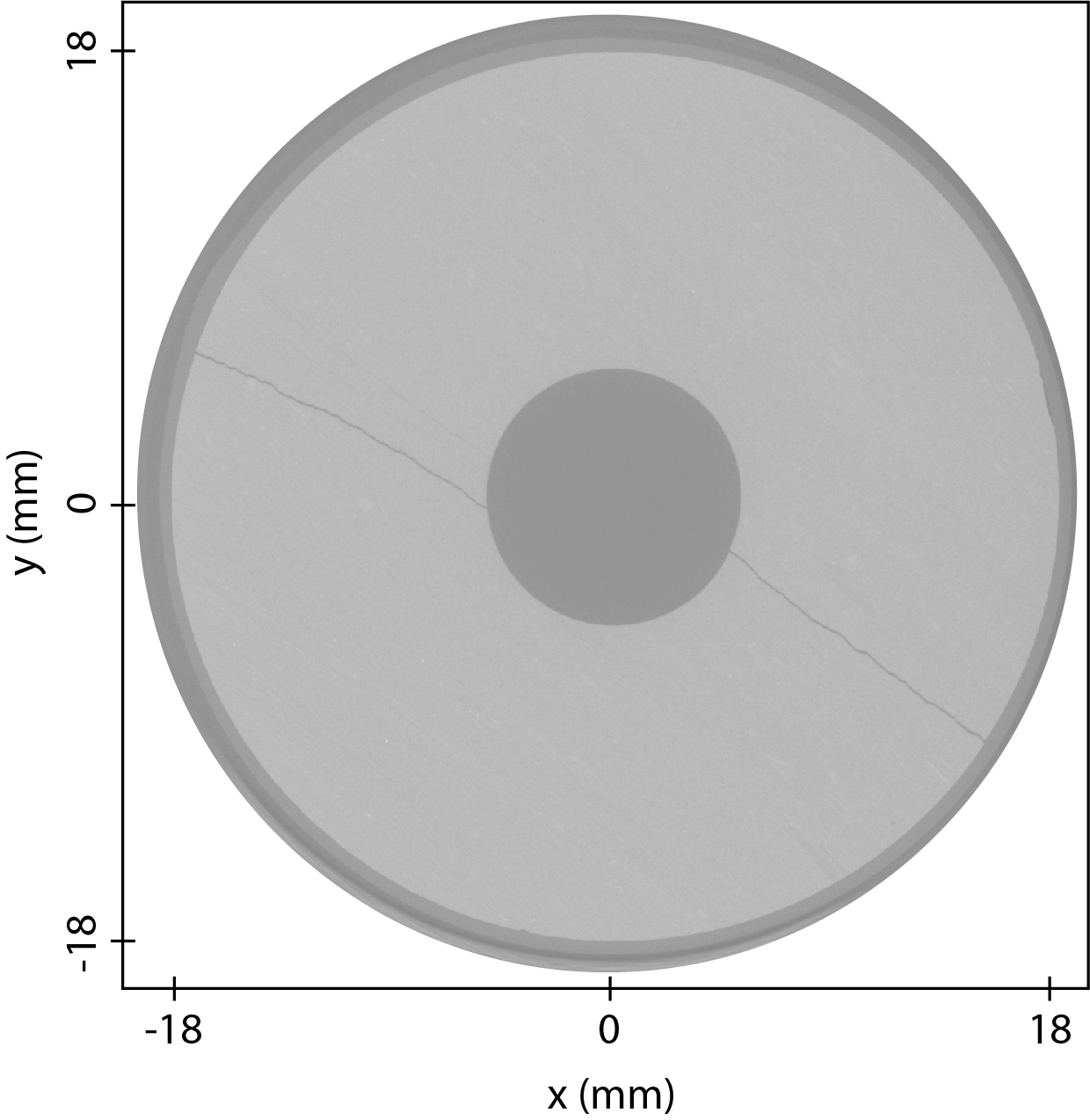


**Supplemental Figure 2:** Micro X-ray Computed Tomography image of sample 132 (from which the flow data was measured) indicating the primary twin fracture network.


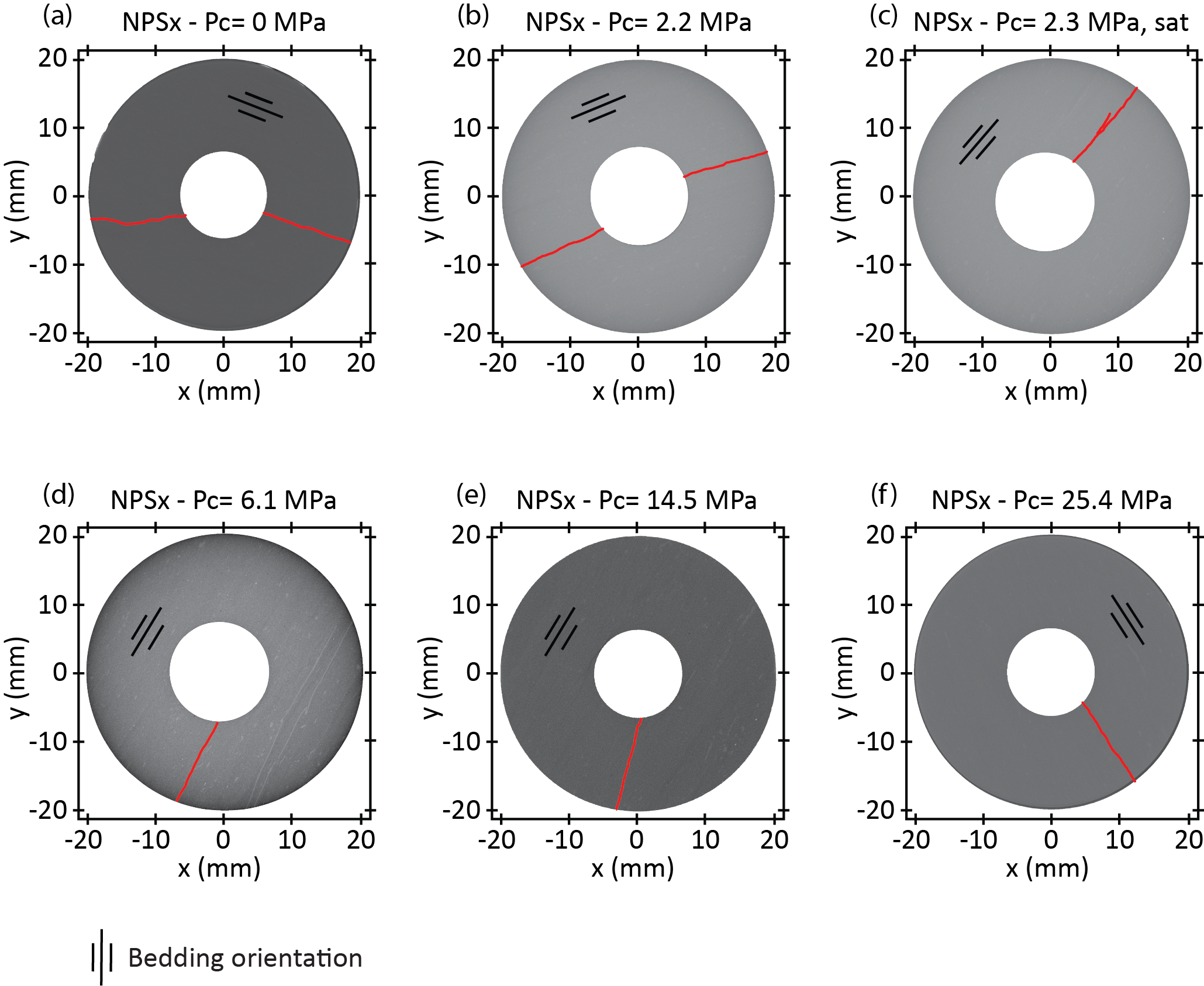


**Supplemental Figure 3:** Micro X-ray Computed Tomography images indicating the fracture network (highlighted in red) developed in Nash Point Shale with the core axis drilled parallel to bedding, black lines indicate the background bedding orientation (after Gehne, 2018). Panels (a) to (f) indicate fracture generated at different confining pressures (Pc), as indicated.
